# Collaborative identification and prioritisation of mental health nursing care process metrics and indicators: a Delphi consensus study

**DOI:** 10.1186/s12913-022-07659-2

**Published:** 2022-03-16

**Authors:** Andrew Hunter, Nora Barrett, Anne Gallen, Gillian Conway, Anne Brennan, Martina Giltenane, Louise Murphy

**Affiliations:** 1 School of Nursing and Midwifery, National University Ireland Galway, Galway, Ireland; 2 Nursing and Midwifery Planning and Development Unit, Health Services Executive, North-West, Dublin, Ireland; 3 Nursing and Midwifery Planning and Development Unit, Health Services Executive, West/Mid-West, Dublin,, Ireland; 4 Nursing and Midwifery Planning and Development Unit, Health Services Executive, North East, Dublin, Ireland

**Keywords:** Quality care metrics, Mental Health Nursing care processes, Delphi survey

## Abstract

**Background:**

The Irish Office of Nursing & Midwifery Services Director (ONMSD) commissioned the development an updated suite of mental health nursing metrics and indicators for implementation in Irish mental health clinical settings. While measuring care processes does offer the potential to improve care quality, the choice of which mental health nursing metrics to measure presents a significant challenge, both in Ireland and internationally. The provision of safe and high-quality mental health nursing care stems from nurses’ expertise, skills and overall capacity to provide recovery focused care across a range of health care settings. Accordingly, efforts to measure what mental health nurses do depends on the identification of those care processes that contribute to mental health nursing practice. This paper reports on the identification, development and prioritisation of a national suite of Quality Care Metrics (QCM), along with their associated indicators, for mental health nursing care processes in Ireland.

**Methods:**

The study was undertaken over four phases; i) a systematic literature review to identify mental health care process metrics and their associated indicators of measurement; ii) a two-round, online Delphi survey of mental health nurses to develop consensus on the suit of mental health nursing care process metrics; iii) a two-round online Delphi survey of mental health nurses to develop consensus on the indicators to be used to measure the agreed metrics; and iv) a face-to-face consensus meeting with mental health nurses and service user representatives to develop consensus on the final suite of metrics and indicators.

**Results:**

Following these four phases 9 metrics and their 71 associated indicators were agreed for inclusion in the final suite of Mental Health Nursing QCM. These metrics are applicable across the life span and the range of mental health nursing health care settings.

**Conclusion:**

The development of this suite of Mental Health Nursing QCM and their indicators represents an opportunity for the measurement of safe and high-quality mental health nursing care for application in Ireland and internationally. This initial development of metrics and indicators should be followed by a rigorous baseline review of QCM uptake and implementation amongst mental health nurses as part of an ongoing evaluation.

## Background

The Health Service Executive (HSE) in The Republic of Ireland, along with many other international health care providers recognise the need for high quality, standardised data collection that quantifies nursing and midwifery care processes [, 1, 2]. While such data can contribute to improved understanding of mental health nursing care processes, the choice of which mental health nursing metrics to measure presents a significant challenge in Ireland and internationally [3].

Contemporary mental health care policy emphasises the need for measurement of the contribution of mental health nurses to patient safety and clinical quality improvement [4], 5, 6]. Currently in Ireland and internationally there very limited research in this area and as such no quantifiable means of establishing the contribution of mental health nursing processes to the outcomes of people in receipt of mental health nursing care across the lifespan and healthcare settings. Notable historical efforts to identify and quantify what mental health nurses do include the development of clinical indicators for mental health nursing in Australia and New Zealand in the 2000s. While not developing metrics and their associated indicators, Skews et al. [7] and O’Brien et al. [8] produced clinical indicators and endeavoured to audit these as a means of comparing and benchmarking practice across mental health settings. It is noteworthy that these studies acknowledged the challenge of capturing the totality of mental health nursing practice and of embedding such measurement approaches. In a discussion on mental health nursing culture Slemon et al. [9] note that mental health nursing practice remains focused on mitigating risk and promoting safety, while lacking effective structures. Indeed, even in these priority areas, where mental health nurses utilise rigorous monitoring and recording as means of risk management the evidence is that current approaches lack efficacy [10].

In response to the identified need the Office of Nursing and Midwifery Services Directorate in Ireland commissioned a national research study to establish a consensus on how nursing and midwifery care processes that should be measured (HSE 2018a). This national study aimed to develop care process metrics and indicators that where possible would align with evidenced-based clinical practice guidelines and standards. This study produced a suite of seven QCM reports that outline these metrics and associated indicators in the healthcare areas of Mental Health, Midwifery, Children’s Community/Public Health, Acute, Older and Intellectual Disability (HSE 2018b). This paper reports the development and prioritisation of a national suite of QCM, and their associated indicators, for mental health nursing in Ireland.

## Methods

This study comprised four discreet phases outlined below. With all phases overseen by a work stream working group made up of mental health nurses from a range of grades, specialities and geographical areas, service user representation and an academic lead (*n* = 30). The work-stream working group members were invited to participate in the quality care metrics project by letter informing them of the study and inviting them to nominate registered mental health nurses of all grades from their area to participate in the study. The group was provided with regular updates by email and teleconference and met face to face four times over the course of the project; before the Delphi study commenced, during the Delphi study and after the Delphi study had completed, to agree the final suite of metrics.

### Phase 1

A systematic literature review to identify mental health nursing metrics that have been used in this area and the indicators for same. The following inclusion criteria were applied; i) Participants: registered nurses or midwives working in any of the seven identified work-streams, or persons in receipt of nursing or midwifery care from eligible work streams; ii) Exposure: relating to nursing or midwifery quality care processes (metrics or indicators). For the purposes of this study a quality care process metric is defined as a quantifiable measure that captures quality in terms of how (or to what extent) nursing or midwifery care is performed in relation to an agreed standard. A quality care process indicator is defined as a quantifiable measure that captures what nurses or midwives are doing to provide care in relation to a specific tool or method (Foulkes et al. 2011 [11]); iii) Outcomes: measurable quality process or processes in use or proposed for use; iv) Type of study: any.

### Phases 2 and 3

A two-round online Delphi survey of mental health nurses to develop consensus on metrics to be prioritised and a two-round online Delphi survey of mental health nurses to develop consensus on indicators for the prioritised metrics. At the end of the first two rounds, the metrics were identified and at the end of Round 3 and 4, the indicators for those metrics were identified. A purposeful convenience sample of mental health nurses working in the mental health services in Ireland were invited to partake in the Delphi survey. Inclusion criteria for the study included any registered mental health nurse working in the HSE mental health services in Ireland, with the ability to give informed consent. Exclusion criteria for the study included any registered mental health nurse not working in the HSE mental health services in Ireland. There were 1102 responses across four Delphi survey rounds.

All mental health nurses who met the inclusion criteria for the study were invited to participate in the study following a comprehensive national advertising campaign via posters, information sheets, word of mouth and presentations at national nursing conferences and events. Participants indicated their interest in the study via email to the research team.

The Delphi data collection and analysis provided group consensus on what metrics (Rounds 1 & 2) and associated indicators (Rounds 3 & 4) should be used. Open fields were provided in Rounds 1 and 3 to allow participants to comment on and suggest additional potential metrics and indicators. Responses to each round were collated then redistributed to participants for further comment in successive rounds. Each round had a response closing date of 21 days after the date of invitation. E-mail reminders were sent to anyone who did not respond by day 7 from the date of invitation. Numbers of participants for each round of the Delphi are presented in Table 1.

**Table 1:** Nursing and midwifery judgement framework tool

Domain	Description
Process Focused	The metrics/indicator contributes clearly to mental health nursing care processes
Important	The data generated by the metric/indicator will likely make an important contribution to improving mental health nursing care processes
Operational	Reference standards are developed for each metric or it is feasible to do so. The indicators for the respective metrics can be measured
Feasible	It is feasible to collect and report data for the metric/indicator in the relevant setting

Modified from: eRegistries indicator evaluation tool [12]

### Phase 4

Face-to-face consensus meeting with key stakeholders to review the findings and build consensus on the final suite of metrics and indicators. Phase 4: Face-to-face meeting with key stakeholders to build consensus on final metrics and indicators.

This phase comprised of a face-to-face meeting with attending members of the work stream working group (n = 20). The work-stream group members were invited to review and build consensus on the quality care process metrics and indicators developed from the Delphi surveys. Participants were provided with a Nursing and Midwifery Judgement Framework Tool adapted from Flenady et al. [12] to guide their determination on metric/indicator inclusion in the final suite of mental health QCMs (see Table 2).

**Table 2:** Overall Delphi responses by grade and role

Grade /Role	Round 1 (*n*)	Round 2 (*n*)	Round 3 (*n*)	Round 4 (*n*)
Staff nurse	57	41	41	22
Clinical nurse manager (1)^a^	6	3	7	2
Clinical nurse manager (2)^b^	76	68	75	47
Clinical nurse manager (3)^c^	22	17	9	10
Assistant Director of Nursing	26	29	27	18
Director of Nursing	1	3	4	3
Community Mental Health Nurse	33	26	22	18
Nurse Practitioner/Registered Nurse Prescriber	5	5	3	2
Clinical Nurse Specialist	0	22	0	0
Clinical Placement Coordinator	0	9	0	0
Other	64	10	45	31
**Total**	**290**	**233**	**233**	**143**

^a^Responsible for the management and delivery of care to the optimum standard within the designated area of responsibility. Generally reports to the Clinical Nurse Manager 2

^b^Responsible for the management of a nursing team and the service delivery within a specific area. Generally reports to a Clinical Nurse Manager 3 or Assistant Director of Nursing

^c^Usually responsible for more than one clinical area within the organisation. The role incorporates resource management and the continuing professional leadership of nursing teams. Reports to the Assistant Director or Director of Nursing

### Ethics

Ethical approval was granted by the Research Ethics Committee National University of Ireland Galway. All potential participants received a study information sheet, which outlined the purpose of the study, the risks and benefits of participation, confirmed participation was entirely voluntary and the likely time commitment. They were also informed they could ask questions at any point and given contact details to that end. All participants had to explicitly indicate their consent to participate by clicking on the ‘I agree’ button at the end of the online participant information sheet before accessing the survey. For phase 4, consensus meeting participants were given a participant information leaflet. Written consent to participate was then obtained at the meeting.

## Results

### Systematic review

The literature search was undertaken as a national collaboration across all 7 work streams (Devane et all 2019 [13], Doody et al. 2019 [14], Murphy et al. 2019 [15]). The aim of the review was to identify quality care process metrics and their associated indicators, and to identify the current evidence base.

Eight databases were systematically searched including: Pubmed, Embase, PyscINFO, ASSIA, Cumulative Index to Nursing and Allied Health Literature (CINAHL), Cochrane Database of Systematic Reviews (CDSR), Cochrane Central Register of Controlled Trials (CENTRAL), and Database of Abstract of Reviews of Effects (DARE).

To maintain contemporary relevance the search was undertaken between 1st January 2007 and 1st January 2017, in English language where full text were available. For this purpose a systematic review procedure was adapted using the search terms nurs*:ab,ti OR midwi*:ab,ti AND (‘minimum data set’:ab,ti OR indicator*:ab,ti OR metric*:ab,ti OR ‘quality measure*’:ab,ti) AND [english]/lim AND [2007–2017]/py. The search was not limited to study design but widened to comprise all types of sources including grey literature.

**Fig. 1:**
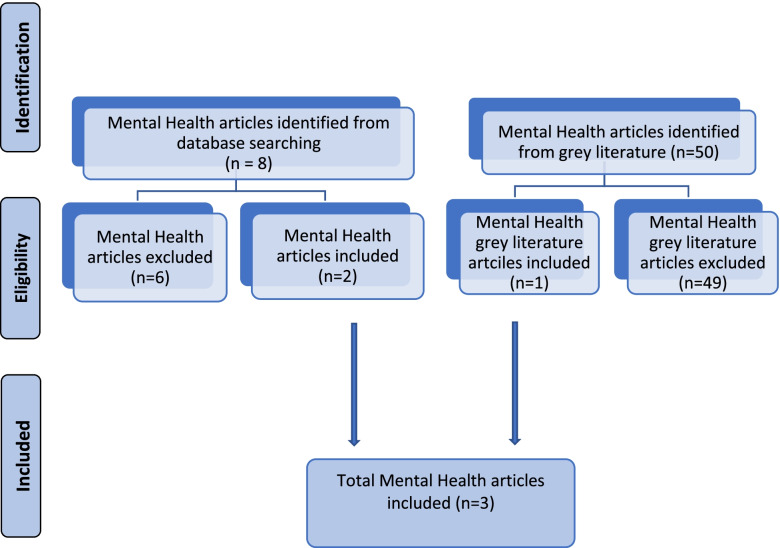
Search and selection flow diagram

Covidence software (Cochrane 2016) was utilised to manage the retrieved studies. After duplicates were removed, each title was reviewed independently by at least two members of the national academic teams. Disputes were settled by discussion and negotiation. At full text screening, any included studies were tagged to the specific work-streams. Full-text studies relevant to each work-stream were then reviewed by two reviewers (NB and AH for mental health nursing) from the appropriate work-stream. The complete process flow diagram for the systematic literature review is presented in Fig. 1. Search and selection flow diagram.

### Systematic review findings

The search conducted across eight databases resulted in 15,304 citations. Following removal of duplicates, 7,524 unique references were identified. Following title and abstract screening, 218 sources were retained, including those identified from hand searching and the grey literature, for full-text screening. Following full text screening, 112 sources were included upon the basis that they met the study’s inclusion criteria. These sources were reviewed for reference to mental health nursing care processes and quality processes (see Fig. 1). Eight studies were identified as relevant to mental health nursing. Two researchers reviewed these independently for quality and content. Disagreements were resolved between the reviewers and a third reviewer consulted if required. Two from the database search [16, 17] both of which referred to measurable approaches to mental health screening, undertaken by mental health nurses. From the grey literature, the Judgement Support Framework [18] a guidance document designed so support quality in mental health care processes implementation and monitoring.

Following the systematic review process, the Mental Health Work Stream Working Group met to discuss the potential metrics extracted from the systematic literature review. At this stage it was agreed that, the pre-existing suite of mental health nursing care process metrics [19] be included in discussion for possible inclusion. Following this discussion, 16 potential mental health nursing metrics were agreed for inclusion in Round 1 of the Delphi survey: 1. Medication Storage and Custody, 2. Management of Controlled Drugs (MDA Drugs), 3. Medication Administration, 4. Ensuring Correct Prescription of Medication, 5. Assessment and Personal Details, 6.Nursing Care Plan, 7.Nursing and Midwifery Board of Ireland (NMBI) Record Keeping, 8. Provision of Required Information for Service Users & Carers, 9. Discharge Planning, 10. Service User Experience, 11. Screening and Evaluation of Mental Health Needs, 12. Use of psychotherapy/nonpharmacological therapeutics i.e. talk therapies, 13. Use of psychiatric medications side effect profile, 14. Care of the dying, 15. Communication of Healthcare Team, 16. Therapeutic Communication with service users.

### Phases 2 and 3 The Delphi consensus process

#### Delphi-round 1

All those who expressed interest in participating were sent email invitations from SurveyMonkey® to participate. A web link was also created as an additional data collector. Round 1 of the Mental Health Metrics Delphi was launched on the 6^th^ of June, 2017 and remained open for 21 Days. The Delphi survey consisted of consent to participate via an: *I agree* or *do not agree* button as well as questions regarding demographics. Participants were asked to rate each of the 16 included metrics from 1–9 (1-Not Important, 9-Very Important). SurveyMonkey® participants were updated weekly according to both Community Health Organisation (CHO) area and nursing grade and circulated to project officers and directors of mental health nursing to track participation rate. Following removal of duplicates and inclusion of only those who provided an email address, there were a total of 290 participants in Round 1.

#### Delphi round 2

All who expressed interest and whose responses provided data from Round 1 were sent email invitations via SurveyMonkey® to participate in Round 2 of the Mental Health Metrics Round 2 Delphi. Participants were also sent confidential emails prior to the start of Round 2 with PDF copies of their individual Round 1 survey responses to allow them to re-rate the metrics based on both their responses and the group’s responses. Round 2 of the Delphi was launched on the 11^th^ of July 2017 and remained open for 21 Days. Following removal of duplicates and inclusion of only those who provided an email address there were a total of 233 participants. Round 2 participants were asked to rate 20 proposed metrics that had either been maintained from Round 1, added from open fields in Round 1 of re-worded following review by the work stream working group.

#### Delphi round 3

This round of the Delphi survey was open to new participants that had not previously participated in Round 1 or Round 2. These participants were asked to rate 80 potential indicators that could be used to measure the seven metrics retained from Rounds 1 and 2. Email invitations via SurveyMonkey® were sent to those who had previously completed Round 1 or Round 2 in addition to any new expressions of interest. Round 3 of the Delphi was launched on the 22^nd^ of August and remained open for 21 days. Following removal of duplicates and inclusion of only those who provided an email address there were a total of 233 participants.

#### Delphi round 4

Email invitations for Round 4 of the Delphi survey were only sent to those who provided data in the Round 3 survey. Participants were also sent confidential emails prior to the start of Round 4 with PDF copies of their individual Round 3 survey responses to allow them to re-rate the indicators based on both their responses and the group’s responses. Round 4 of the Delphi was launched on the 3^rd^ of October 2017 and remained open for 21 Days. Following the removal of duplicates and inclusion of only those who provided an email address there were a total of 143 participants.

### Delphi rounds data analysis

As noted participants across the Delphi rounds were asked to indicate the importance of the metrics and indicators using a 9-point Likert scale. At the completion of Delphi round 2 (metrics) and Delphi round 4 (indicators) consensus on inclusion was determined where 70% or more participants rated the metric or indicator as 7 to 9 and less than 15% of participants rated the metric as 1 to 3. These criteria were used consistently across the four rounds to best represent the participants’ judgements over the rounds [20]. The 70% importance rate is also consistent with approaches to developing core outcome sets in routine healthcare http://www.comet-initiative.org/.

### Phase 4

Consensus meeting participants voted on each metric and indicator as a yes or no to be included using the Poll Everywhere App. Metrics and indicators were required to receive a vote of 70% or higher to be included for the final suite in mental health quality care metrics. Follow-up discussions and multiple rounds of voting were used where necessary. Following this process, the final agreed suite of nine mental health nursing process metrics and their indicators were finalised (see Table 3):

**Table 3:** Final agreed metrics (*n* = 9) and indicators (*n* = 71) with Delphi round 4 consensus percentages

METRIC	INDICATOR	FINAL DELPHI ROUND 4 CONSENSUS PERCENTAGE
**ASSESSMENT**	1 Presenting Complaints/Reasons for admission/attendance is recorded and the admission date and times are recorded	98.66%
	2 The service user's name/date of birth and Healthcare Record Number are on each page/screen	95.30%
	3 Initial assessment includes contact details for family member/carer	98.66%
	4 There is a documented reason if the service user refuses to give next of family member/carer details	81.21%
	5 There is documented evidence of discharge planning is recorded from admission	82.55%
	6 There is documented evidence of service user consent for family member/carer involvement in care and communication	90.60%
	7 The service user is involved in all aspects of his/her assessments e.g. falls, risks, neglect etc. as per local policy	92.62%
	8 It is documented that the mental health service, with the service user's informed consent has involved other named service providers in their assessment if required	98.66%
**CARE PLAN**	1 All entries are in chronological order	94.63%
	2 Nursing interventions are individualised and include nurse's title, name, signature, the date and time	91.28%
	3 All records are legible, in permanent black ink	95.97%
	4 Student entries are countersigned by the supervising nurse	92.62%
	5 There is documented evidence that the service user is involved in a co- production of their nursing care plan	93.96%
	6 Any alterations in nursing documentation are as per Nursing and Midwifery Board of Ireland (NMBI) Guidelines	88.59%
	7 There is documented evidence that the nursing care plan has been reviewed on a regular basis, as defined by the individual clinical area	83.89%
	8 Any abbreviations/grading systems used are from a national or locally approved list/system	77.18%
**MANAGEMENT OF RISK**	1 All entries are in chronological order	97.32%
	2 Nursing interventions are individualised and include nurse's title, name, signature, the date and time	97.99%
	3 All records are legible, in permanent black ink	97.99%
**MANAGEMENT OF VIOLENCE AND AGGRESSION**	1 There is documented evidence that all incidents of violence and aggression are recorded	98.66%
	2 There is documented evidence that timely and appropriate post- incident debriefing has occurred for service users	89.26%
	3 There is documented evidence in the nursing care-plan of nursing responses/interventions to violent and aggressive incidents and risk	91.28%
**PHYSICAL HEALTH AND WELLBEING**	1 There is documented evidence that that medical history is recorded in the service user’s notes	93.92%
	2 The allergy status is clearly identifiable on relevant nursing documentation	97.30%
	3 There is documented evidence of an ongoing physical health assessment from admission/referral	89.26%
	4 There is documentary evidence that identified	83.22%
**SERVICE USER EXPERIENCE**	1 Were you given information about this service?	94.48%
	2 Were you introduced to the nurse or nurses responsible for your care?	84.83%
	3 Do you know the names of your nursing team?	78.62%
	4 Have you received information from your responsible nurse on how to manage symptoms of your illness?	97.24%
	5 Has your medication and any potential benefits/side effects been explained to you by your responsible nurse?	94.48%
	6 Have you got the relevant information on who to contact in times of a crisis?	97.24%
	7 Were you involved in developing your nursing care plan?	94.48%
	8 Were you offered a copy of your care plan?	82.07%
	9 Have you been offered the opportunity to have your family/carer involved in your care?	93.10%
	10 Are you offered 1:1 nursing time as indicated in your care plan?	85.52%
	11 Has information been offered on organised activities/groups in your area?	91.72%
	12 Do the activities/groups offered support you in your recovery process?	89.66%
	13 Is there the opportunity for access to outside space?	91.03%
	14 Can you access fresh drinking water?	89.66%
**RECOVERY BASED CARE**	1 The service user has been informed of / offered peer support to aid in their recovery	77.93%
	2 The nurse has documented evidence that the service user has access to a recovery-based programme	88.28%
	3 There is documented evidence that the service user is involved in all aspects of his/her recovery planning including discharge planning	96.64%
	4 There is documented evidence in the nursing care plan that the nurse has provided information about voluntary services that may help service users in their recovery process	69.13%
**NURSING COMMUNICATION**	1 There is evidence in the clinical notes that a nurse has communication with the service user as per care plan	93.79%
	2 The nurse has offered the service user has received information regarding their rights	93.79%
	3 There is documented evidence in the nursing care plan that the nurse has offered the service user information on advocacy services and how to access them	83.45%
	4 There is documented evidence to support the coordination of nursing care on transfer or discharge	95.17%
	5 There is documented evidence that the service user's communication style and preferences are recorded in the nursing notes	77.85%
**MEDICATION MANAGEMENT**	1 There is documented evidence in the nursing notes that medication side effects are assessed by the nurse	94.48%
	2 A registered nurse is in possession of the keys for Medicinal Product Storage	95.10%
	3 All Medicinal products are stored in a locked cupboard or locked room	95.10%
	4 All medication trolleys are locked and secured as per local organisational policy and open shelves on the medication trolley are free of medicinal products when not in use	98.60%
	5 A current Drug Formulary is available on all Medication Trolleys	98.60%
	6 Misuse Drug Act (MDA) drugs are checked & signed at each changeover of shifts by nursing staff. ( member of day staff & night staff)	92.31%
	7 Two signatures are entered in the MDA Drug Register for each administration of an MDA drug	94.41%
	8 The MDA Drug cupboard is locked and keys for MDA cupboard are held by designated nurse	97.90%
	9 MDA drug keys are kept separate from other medication keys	95.10%
	10 The individual’s prescription documentation provides details of individual’s legible name and health care record number	95.10%
	11 The Individuals’ identification band has correct and legible name and healthcare record number and/or photo ID if in use	98.60%
	12 The allergy status is clearly identifiable on the front page of the prescription chart	98.60%
	13 Prescribed Medication not administered have an omission code entered	92.31%
	14 The generic name is used for each drug prescribed	94.41%
	15 The date of commencement of the most recent prescription is recorded	97.20%
	16 The Prescription is written in block letters	90.91%
	17 The correct legible dose of the drug is recorded with the correct use of abbreviations	98.60%
	18 The route and/or site of Administration is recorded	91.61%
	19 The frequency of administration is recorded & correct timings indicated	98.60%
	20 The minimum dose interval and/or 24 h maximum dose is specified for all “as required” or PRN drugs	98.60%
	21 The prescription has a legible prescriber’s signature (in ink)	90.21%
	22 Discontinued drugs are crossed off, dated and signed by a person with prescriber authority	97.20%

## Discussion

The 9 metrics and 71 indicators presented in Table 3, represent the main findings of this study. The metrics and indicators included in the final suite show considerable synergy with literature indicating areas where mental health nurses and service users have identified the need to develop the knowledge, skills and practice of mental health nurses [21]. By identifying prioritised areas where mental health nursing processes can be measured this research contributes to efforts to quantify and improve practices that mental health nurses, service users and carers representatives value. This understanding of these stakeholders’ views on key areas of nursing process indicate potential areas for the development of mental health nursing practice and education.

It should be noted that metrics as applied to health care practices arise from the world of business where metrics were devised as means of setting targets and measuring attainment of these [22]. Pencheon [23] suggests that within healthcare settings, metrics as measured by their indicators illustrate areas of practice performance and the degree to which expectations are being met. Hence, these can be used as a quality control measure in practice. Typically, metrics and their associated indicators are used to show benchmarking or attainment against agreed standards, with a view to ensuring and improving the quality of care [, 24–26]. It should be noted that while there are clear potential benefits to the implementation of metrics, there is ongoing concern that care processes, in this case the day to day business of mental health nursing cannot be adequately measured [3]. The concern being that the interpersonal day to day activities delivered by mental health nurses are hard to measure and that mental health nurses do not work alone making their activities as distinct from multi-disciplinary team activities hard to isolate and quantity [27].

In this study, despite the lack of applicable evidence based metrics and indicators identified from the systematic review, the grey literature identified valuable starting points for the deliberations of the work-stream working group, in identifying areas of importance to mental health nursing in the Irish context. The literature sources that were identified ranged from full procedure guidelines with some underpinning evidence through to checklists of areas pertaining to practice to be measured.

Central to the conduct of this research and complimented by the rigorous research process outlined is the level of engagement with service user and family representatives and a broad cross-section of mental health nurses across grades, practice settings and geographical areas in Ireland. This ensured that mental health nursing QCMs and associated indicators arose from genuine consensus. Crucially there was service user and family representation from the outset, across the work stream working group deliberations through to the final Phase 4 consensus meeting, ensuring maximum possible involvement in the ongoing research. This approach reflects the policy emphasis in Ireland that mental health service provision should arise from genuine partnership between service users and service providers [28–30].

Despite the lack of applicable evidence identified from the systematic review every effort was made to ensure the quality of the mental health nursing metrics and indicators included in the final suite. The evaluation tool used identified four key attributes of metrics and indicators these being process focused, important, operational and feasible. The rigorous research design employing 4 Phases; systematic literature review, online Delphi survey of mental health nurses and consensus meeting with key stakeholders, means that the finally agreed metrics and indicators can be considered to be process focused with clear applicability mental health service users and mental health nurses in practice. Importantly, as noted in the systematic review section, not all of the agreed metrics and indicators have supporting clinical standards and an evidence base although they do have a strong practice evidence base. This understanding specifically impacts on feasibility, the fourth component of the judgement framework tool. This suggests that there is a need for robust piloting and evaluation of these metrics and indicators before rolling out service wide to identify potential unintended impacts and barriers [31]. Consequently, this initial development of metrics and indicators should be followed by a rigorous baseline review of QCM uptake and implementation amongst mental health nurses as part of an ongoing evaluation.

## Conclusion

Achieving consensus on what mental health nurses do and how best to measures those processes in an important element of ongoing efforts to improve the safety and quality of mental health nursing practice. Having achieved its aim in producing a suite of mental health nursing metrics and indicators, there is now the opportunity to incorporate routine measurement into ongoing efforts to improve the quality of mental health nursing care. Any implementation of these metrics and indicators could provide valuable evidence of mental health nurses contribution to safe, quality, care processes.

## Data Availability

The datasets used and/or analysed during the current study available from the corresponding author on reasonable request.
